# Size-Controlled Transformation of Cu_2_O into Zero Valent Copper within the Matrix of Anion Exchangers via Green Chemical Reduction

**DOI:** 10.3390/polym12112629

**Published:** 2020-11-09

**Authors:** Irena Jacukowicz-Sobala, Ewa Stanisławska, Agnieszka Baszczuk, Marek Jasiorski, Elżbieta Kociołek-Balawejder

**Affiliations:** 1 Department of Industrial Chemistry, Wrocław University of Economics and Business, ul. Komandorska 118/120, 53-345 Wrocław, Poland; ewa.stanislawska@ue.wroc.pl (E.S.); elzbieta.kociolek-balawejder@ue.wroc.pl (E.K.-B.); 2 Department of Mechanics, Materials Science and Engineering, Wrocław University of Science and Technology, ul. Smoluchowskiego 25, 50-370 Wrocław, Poland; agnieszka.baszczuk@pwr.edu.pl (A.B.); marek.jasiorski@pwr.edu.pl (M.J.)

**Keywords:** zero valent copper, Cu^0^-containing hybrid anion exchanger, Cu_2_O reduction, ascorbic acid as reducer

## Abstract

Composite materials containing zero valent copper (ZVC) dispersed in the matrix of two commercially available strongly basic anion exchangers with a macroreticular (Amberlite IRA 900Cl) and gel-like (Amberlite IRA 402OH) structure were obtained. Cu^0^ particles appeared in the resin phase as the product of the reduction of the precursor, i.e., copper oxide(I) particles previously deposited in the two supporting materials. As a result of a one-step transformation of preformed Cu_2_O particles as templates conducted using green reductant ascorbic acid and under mild conditions, macroporous and gel-type hybrid products containing ZVC were obtained with a total copper content of 7.7 and 5.3 wt%, respectively. X-ray diffraction and FTIR spectroscopy confirmed the successful transformation of the starting oxide particles into a metallic deposit. A scanning electron microscopy study showed that the morphology of the deposit is mainly influenced by the type of matrix exchanger. In turn, the drying steps were crucial to its porosity and mechanical resistance. Because both the shape and size of copper particles and the internal structure of the supporting solid materials can have a decisive impact on the potential applications of the obtained materials, the results presented here reveal a great possibility for the design and synthesis of functional nanocrystalline solids.

## 1. Introduction

The development of nanoscience and nanotechnology in the past two decades has opened up new areas for the use of copper in the form of ultrafine particles with specific chemical, electrical, optical, and thermal properties dependent mainly on their size and shape. Because of their extremely small size, high surface energy, and large surface area/volume ratio, Cu nanoparticles (CuNPs) have different (e.g., physicochemical and biological) properties than those of their bulk metallic form [1,2,3]. Owing to their antimicrobial activity against a wide range of microorganisms, including multidrug-resistant organisms and fungi, CuNPs have been a strong focus of research on health-related processes [4,5]. They have also attracted considerable attention in the fields of heterogeneous catalytic reactions, including industrially important processes such as reduction of aromatic nitrocompounds, azide-alkyne cycloaddition, coupling reactions, advanced oxidation processes, and also photo- or electrocatalytic processes [2,3,6,7,8,9,10].

CuNPs have been synthesized using a variety of techniques. Among them, chemical reduction is the most frequently used due to its simplicity, limited equipment requirements, low cost, and possibility of synthesizing copper nano/microstructures with various morphologies. In these reactions, the source of copper most often is a cupric salt reduced by hydrazine or sodium borohydride. By selecting a reducing agent and varying the reaction conditions, one can control the size and shape of the obtained copper particles [11,12,13,14,15]. Unfortunately, the latter, due to van der Waals forces, tend to be rather unstable in solution, which leads to large agglomerates during the dispersion processing (preparation and storage). Moreover, copper nanoparticles are susceptible to oxidation when exposed to air, so the synthesis of stable products requires the presence of stabilizers. In order to protect CuNPs against their sticking and oxidation, surface-active agents, such as surfactants, dissolved polymers, and organic ligands (known as capping agents), can be used in aqueous media as dispersants and surface modifiers to yield highly stable, well-dispersed particles [13,16,17]. Another promising strategy concerning the stability of CuNPs is their immobilization on supporting solid materials, both of natural origin (cellulose, cotton, paper, chitosan, carbon active) and synthetic polymers (zeolite, graphene oxide, cation exchangers) [18,19,20,21,22,23].

The group of polymeric/inorganic composites based on ion exchangeable reactive polymers, also called hybrid ion exchangers (HIXs), is significantly prospective due to the presence of different active components such as a metal deposit and positively or negatively charged functional groups of supporting polymers [24,25,26,27,28]. The dispersion of NPs within the matrix of such a host material not only prevents their agglomeration and ensures contact between the reagents, but it also, owing to many possible combinations of hybrid polymers’ components, enables one to develop multifunctional materials, which frequently exhibit synergetic effects. The physical form of spherical beads of HIXs makes them suitable (as opposed to parent NPs) for use in dynamic conditions in fixed bed column systems. In turn, the functional groups of the polymer modify their activity by the preconcentration of the reagents in the solid phase of the HIXs. Furthermore, functional groups may also have specific catalytic, stabilizing, or biocidal properties (in addition to CuNP activity) [1,29,30,31].

Copper-containing hybrid ion exchangers are synthesized based on sulfonic and carboxylic cation exchangers useful as functional materials for water deoxygenation. The introduction of metallic copper into cation exchangers containing negatively charged functional groups (sulfonic, carboxylic) is not difficult because these groups in the ion exchange reaction quantitatively bind Cu^2+^ ions from the solution. When a cation exchanger in the Cu^2+^ form was treated with a reducing agent solution (hydrazine, sodium dithionite), Cu^0^ particles precipitated in its matrix [32,33,34]. Considering the multifunctionality of the hybrid polymers containing copper deposit, the choice of an anion exchanger as a supporting material is a very promising challenge. This choice is particularly interesting due to the presence of quaternary ammonium groups, which not only may be involved in preconcentration of negatively charged reagents in the solid phase of the ion exchanger but also show antimicrobial activity (in contrast to the functional groups of the cation exchangers) and can enhance the biocidal efficiency of the obtained CuNP-containing composites [35]. However, it is impossible to introduce Cu^0^ particles into the anion exchange matrix in an analogous way to the synthesis of Cu^0^-containing cation exchangers. The positively charged quaternary ammonium functional groups repel Cu^2+^ ions. Therefore, using the anion exchanger as a supporting material is challenging and requires an unconventional strategy.

In considering ways of obtaining anion exchangers with metallic copper, we came to the conclusion that this aim could be achieved most easily using anion exchangers containing Cu_2_O, as described in our previous studies [36]. This material was selected as a candidate for the production of HIXs containing metallic copper because it contains low oxidation copper atoms, which can be relatively easily reduced at room temperature with ascorbic acid [15,37,38,39,40]. Strong reducers, such as hydrazine and NaBH_4_, are not only toxic and flammable chemicals, but they also release a gaseous product in the reaction, which could be unbeneficial when the substrate subjected to the reaction is an anion exchanger in the form of a porous solid. Low temperature during reduction is necessary due to low thermal resistance of anion exchangers (up to 60–70 °C).

Therefore, the aim of this study is to transform the Cu_2_O deposited in the anion exchanger’s matrix to Cu^0^ through a simple and environmentally benign reaction with ascorbic acid as the reducer. More precisely, our objectives are (1) to determine whether the transformation of Cu_2_O to Cu^0^, which easily proceeds in (bulk) aqueous media, would also occur in mild conditions in the solid phase of anion exchangers; (2) to examine the influence of reaction conditions such as pH < 7, time, temperature, and intermediate drying on the efficiency of Cu_2_O conversion into Cu^0^ within the polymeric matrix; and (3) to determine whether the type of supporting polymer structure (macroporous or gel-type) influences the distribution, size, and shape of deposited CuNPs. The present study is unique since its aim is not simply to introduce a deposit into the anion exchanger phase but to determine how as a result of a chemical reaction, the introduced deposit changes into a deposit characterized by a different chemical structure (and specific physicochemical properties). Such composite materials—zero valent copper (ZVC) immobilized and stabilized in the matrix of anion exchangers (An/Cu)—deserve special attention due to their unique method of synthesis, unexpected physicochemical properties, and potential applicability in multidisciplinary fields connected with environmental protection.

## 2. Materials and Methods

### 2.1. Materials

The polymer supports for Cu_2_O and Cu^0^ were Amberlite IRA 900Cl (M/An) and Amberlite IRA 402OH (G/An)—two commercially available anion exchange resins produced by The Dow Chemical Co. (Midland, MA, USA). All the chemicals used in this study, including ascorbic acid(+) (POCh Gliwice, Poland), CuCl_2_∙2H_2_O (Chempur, Piekary Śląskie, Poland), HNO_3_ 65% (PPH Stanlab, Lublin, Poland), NaOH, HCl 35%, and cuprizone 98% (Alfa Aesar, (Ward Hill, MA, USA)), were of analytical grade. All the solutions were prepared using deionized water.

### 2.2. Synthesis of An/Cu_2_O

The starting anion exchangers were dried at 40 °C for 24 h in a dryer chamber. To transform the functional groups in the ${\mathrm{CuCl}}_{4}^{2-}$ form, a sample of the anion exchanger weighing about 2.0 g was placed in a conical flask and treated with 20 cm^3^ of 0.5 mol dm^−3^ CuCl_2_ in 5 mol dm^−3^ HCl. The reagents were shaken at 20 °C for 1 h (M/An) or for 4 h (G/An). To carry out the reduction reaction, the filtered off (under vacuum) intermediate product was placed in a conical flask, was treated with 40 cm^3^ of the 0.5 mol dm^−3^ ascorbic acid (AA) solution in 2 mol dm^−3^ NaOH, and was shaken at 20 °C for 24 h. After this time, the sediment was removed from the reaction medium through decantation, and the filtered off and washed product (An/Cu_2_O) was dried at 40 °C for 24 h or kept in the wet state [36].

### 2.3. Synthesis of An/Cu^0^

The synthesis of An/Cu^0^ was conducted using two methods depending on the form of starting material An/Cu_2_O—i.e., dried at 40 °C (Method 1) or wet state filtered off under vacuum to moisture content 50 % (Method 2). A sample of An/Cu_2_O containing about 0.5 g dry matter was placed in a conical flash and treated with 20 cm^3^ of the 1 mol dm^−3^ AA solution in water alone or in 1 mol dm^−3^ NaOH (Table 1). The reagents were shaken at 20 °C for 24 h or at 50 °C for 3 h (or for 30 min). After this time, any sediment was removed from the reaction medium through decantation, and the filtered off and washed product (An/Cu) was dried at 40 °C for 24 h.

### 2.4. Characterization

The total content of Cu in An/Cu was determined using inductively coupled plasma atomic emission spectroscopy (Thermo Scientific iCAP 7400, Waltham, MA, USA) after a sample mineralization using the digestion microwave system MARS 5 (CEM Corporation, Matthews, NC, USA). The Cu content in the An/Cu was determined also after dissolving Cu^0^ in nitric acid solution. A sample of An/Cu weighing about 0.2 g was treated with 10 cm^3^ of HNO_3_ (1:3) in a conical flask and shaken for 24 h. After this time, the polymer sample was filtered off and washed with deionized water. The solution was analyzed spectrophotometrically using the cuprizone (bis(cyclohexanone)oxaldihydrazone) method. Analyses were repeated twice, and the standard deviation did not exceed 1.0%. The redox potential values of the reducer solutions were measured with the electrode ERPt-11X1 (Hydromet, Gliwice, Poland).

The synthesized materials were investigated by scanning electron microscopy (SEM), X-ray diffraction, and FTIR spectroscopy. The morphology and elemental composition of the different An/Cu samples were examined with a Hitachi S-3400 N scanning electron microscope (Tokyo, Japan) equipped with an energy-dispersive spectrometry (EDS) microanalyzer. The FTIR spectra of prepared An/Cu samples were measured using a Fourier transform Bruker VERTEX 70 V vacuum spectrometer equipped with an air-cooled DTGS detector (Billerica, MA, USA). The XRD patterns were measured on an X-ray Rigaku Ultima IV diffractometer using a Cu Kα radiation source (λ = 1.5406 Å) operating at 40 kW/40 mA with the range from 10° to 100° in 0.05° steps with an exposure time of 4 s per point.

To analyze the surface and pore parameters of the M/An/Cu and M/An/Cu-2, the vacuum filtered off product, after washing with deionized water, was dried by applying thermally drying or freeze-drying techniques. The freeze-drying process was conducted by treating the materials at −80°C for 24 h, followed by drying at a pressure of 0.02 mbar for 24 h using a Labconco FreeZone Laboratory Freeze Dryer 4.5 L. The porous characteristics of the samples were determined by the low temperature N_2_ adsorption–desorption at 77 K using an ASAP 2020 Micromeritics porosimetry analyzer; mercury intrusion porosimetry was conducted on a Micrometrics AutoPore IV 9510 analyzer.

## 3. Results and Discussion

### 3.1. Optimization of ZVC Content within the Matrix of Anion Exchangers

**Scheme 1:**
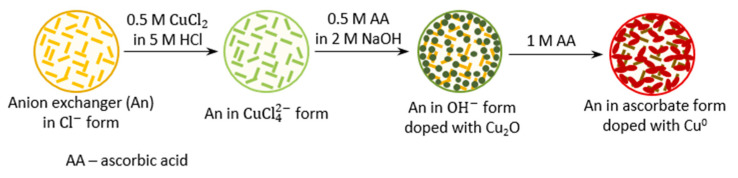
Rout of synthesis of hybrid ion exchangers (HIXs) containing metallic copper deposit.

The chemical deposition of the Cu^0^ particles inside a strongly basic anion exchanger represents a multistage process that has three steps (Scheme 1).

In order to accomplish the last transformation and obtain an anion exchanger with metallic copper particles in the resin phase, the following reaction (1) was carried out:


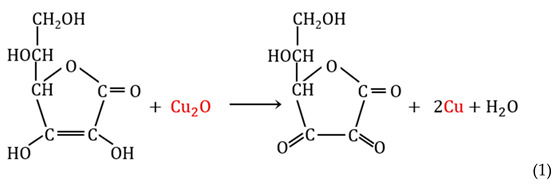


Two commercially available anion exchangers containing the same type and comparable number of strongly basic quaternary ammonium functional groups (3.0 meq g^−1^), but varying in the structure of the poly(styrene/divinylbenzene) skeleton (macroporous Amberlite IRA 900Cl or gel-like Amberlite IRA402OH), were used as the polymeric support for the ZVC. The Cu_2_O deposit was introduced into the anion exchanger matrix in two steps: First, the anion exchanger functional groups were transformed into the ${\mathrm{CuCl}}_{4}^{2-}$ form, and then the intermediate polymeric product was contacted with an alkaline ascorbic acid solution [36]. It was this reducer that in a highly alkaline environment made it possible to practically quantitatively precipitate Cu_2_O into the resin phase (not into the outside of the latter as in the case of glucose). In our previous study, it was shown that a large amount of this deposit could be successfully introduced into both macroreticular and gel-like anion exchangers. The very high Cu_2_O content and the advantageous distribution of the deposit throughout the volume of ion exchanger beads (Table 2) make them ideal substrates for synthesizing even more unique materials, such as metallic copper-doped anion exchangers. Detailed morphological characteristics were described in our previous study [36].

It was reported in the literature that by reducing cupric compounds with ascorbic acid, one can obtain different products, depending on the reaction medium pH. If ZVC is to be obtained, pH < 7.0 should be ensured. Moreover, it has been shown that the lower the pH, the faster Cu^0^ particles form, and the higher the pH, the smaller are the particles [37,38,39,40]. Considering that the reactions described in the literature were conducted in bulk solutions in different conditions, in order to transform Cu_2_O to Cu^0^ in the anion exchanger matrix (i.e., in atypical conditions this is more difficult because of the necessity of diffusing the reagents within the polymeric skeleton of the supporting material), first, the experiments whose results are presented as Method 1 in Table 3 were carried out. Two reaction solutions with pH < 7, but differing in their acidity—namely AA/2, a solution of ascorbic acid alone (with pH ~2), and AA/6, a solution neutralized with an equimolar amount of NaOH (sodium ascorbate, with pH ~6)—were used, and an appropriately long time of contact between the reagents at different temperatures was ensured.

The data in Table 3 indicate that regardless of the reduction conditions, after the reaction all the products contained much copper. This means that the skeletons of both the macroreticular and gel-like anion exchangers retained the deposit undergoing transformation from Cu_2_O to Cu^0^. Even though the Cu content in the An/Cu matrix, amounting to 4.50–7.68%, expressed the actual copper content in the sample, this result did not fully reflect the degree of transformation of Cu_2_O into Cu^0^. The mass of the An/Cu samples was found to be up to 25% greater than that of the initial An/Cu_2_O samples, which was owing to the transformation of the anion exchanger’s functional groups from the hydroxyl into the ascorbate form. It is worth emphasizing here that this form of exchanger functional groups is particularly advantageous due to its antioxidant properties protecting the newly formed copper from undesirable oxidation. Considering the gain in sample mass, one can estimate that in the case of the samples with the highest copper content, the degree of conversion amounted to over 95%, and in none of the cases was it lower than 85%. Thus, it turned out that neither the long shaking of the sample nor the “loosening” of the polymer structure at the elevated temperature nor the character of the reaction medium (ascorbic acid with a low pH or an almost neutral sodium ascorbate solution) resulted in any loss of a significant part of the inorganic deposit, which is difficult to achieve when attempting to obtain such materials as HIXs. However, it should be noted that when M/An/Cu_2_O was the reagent, the reaction medium became slightly cloudy, which was ascribed to the crumbling of the ion exchanger rather than to the passing of the inorganic deposit to the aqueous phase.

### 3.2. Characterization of the Form of Inorganic Deposit

#### 3.2.1. XRD Analysis

**Figure 1:**
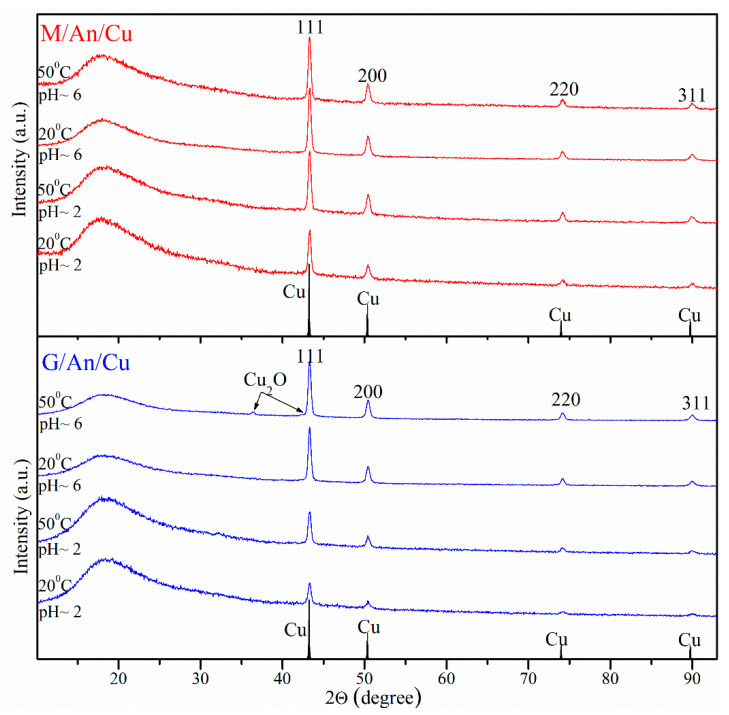
XRD patterns of reduction reaction products M/An/Cu and G/An/Cu.

The content of Cu deposited in the structure of anion exchangers (Table 3) may not totally reflect the content of zero valent copper nanoparticles present in the samples determined after their mineralization. Since both the precursor (Cu_2_O) and product (metallic Cu particles) form crystalline structures, eight samples of hybrid polymers obtained in different conditions were examined by X-ray diffraction analysis. XRD patterns obtained for hybrid anion exchanger samples are shown in Figure 1. The peaks visible on diffractograms of both types of ion exchangers, located at 43.3°, 50.4°, 74.2°, and 90.0° 2Θ, undoubtedly correspond to metallic copper (ICSD collection code: 64699) in the form of a single face-centered cubic cell. A closer analysis of Figure 1 reveals additional low-intensity reflections on the diffraction pattern of the gel HIX sample obtained after the reduction of G/An/Cu_2_O at an elevated pH and temperature (pH ~6, 50 °C). The most intense of the additional peaks is located at 36.4° 2Θ, which indicates that it is associated with the presence of Cu_2_O in the G/An/Cu sample. The presence of residual, unreduced Cu_2_O is probably a consequence of too short reaction time (3 h) in spite of elevated temperature. In contrast, a 3 h duration of the process was sufficient for the reduction of Cu_2_O in the structure of the macroporous anion exchanger due to a significantly better developed porous structure.

As can be seen in Figure 1, in addition to distinct peaks from metallic copper, all diffractograms show an amorphous halo with a maximum at about 18°. The presence of this characteristic very wide peak can be associated with the amorphous polymer anion exchanger [41]. A comparison of diffractograms of macroreticular M/An/Cu samples obtained in different reduction conditions reveals that the ratio of the intensity of the broad polymer peak to the (111) peak of metallic copper is the highest $\left(\frac{I_{amorf}}{I_{\left(111\right)Cu}}>1\right)$ for the sample obtained at low pH and room temperature. In the remaining M/An/Cu samples, the (111) copper peak has a much higher intensity than that observed for the polymer $\left(\frac{I_{amorf}}{I_{\left(111\right)Cu}}<1\right)$, which indicates a significant content of metallic copper in these samples. An analysis of the XRD results of the gel G/An/Cu samples shows that the ratio of the intensity of the broad halo-called amorphous peak to the most intense (111) peak of metallic copper is always higher $\left(\frac{I_{amorf}}{I_{\left(111\right)Cu}}>1\right)$ in samples obtained at lower pH than for samples reduced at pH ~6 $\left(\frac{I_{amorf}}{I_{\left(111\right)Cu}}<1\right)$, regardless of the temperature during the reduction. This indicates a higher content of metallic deposit, regardless of the type of exchanger, in samples reduced at pH ~6. These results are compliant with the total Cu content in the studied hybrid polymers. It may be due to a higher dissociation degree of ascorbic acid at neutral pH (pK_a_ = 4.05) and its electrostatic attraction by positively charged functional groups of the polymer that the diffusion of the reducer into the matrix of anion exchanger was facilitated. In addition, the increase of pH decreases the redox potential of ascorbic acid, which makes it a more powerful reducer at higher pH [36].

#### 3.2.2. FTIR Analysis

**Figure 2:**
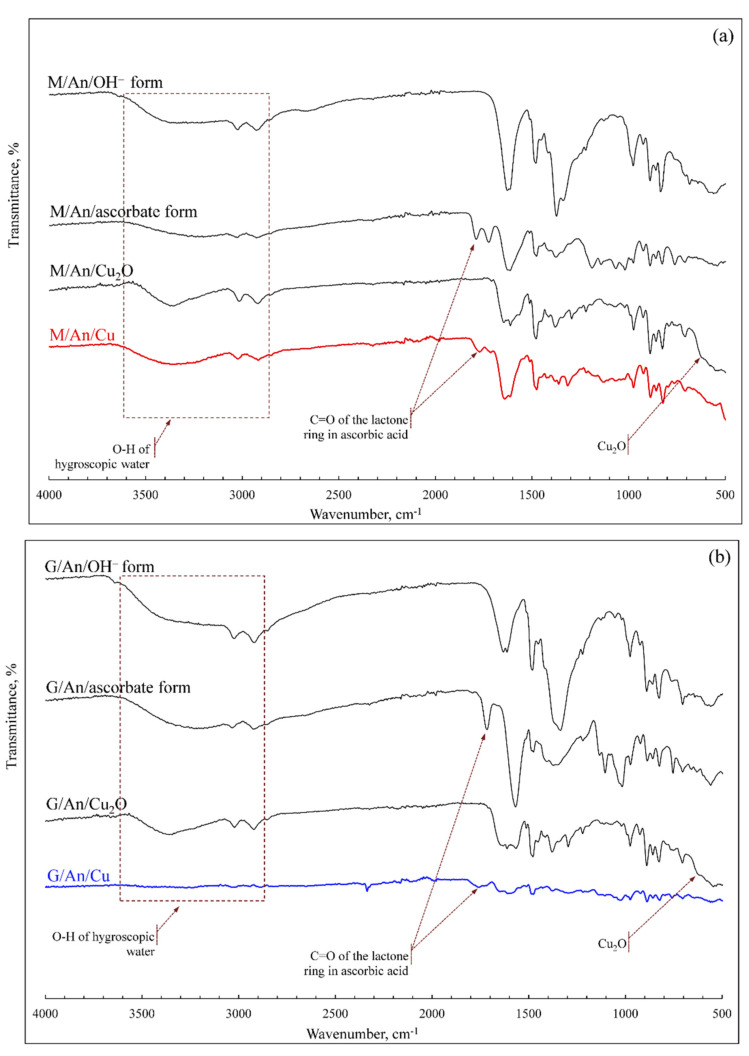
FTIR spectra of virgin anion exchangers in the OH^-^ and ascorbate form: M/An (**a**) and G/An (**b**); starting hybrid polymers containing Cu_2_O: M/An/Cu_2_O (**a**) and G/An/Cu_2_O (**b**); and final products containing Cu^0^ deposit: M/An/Cu (**a**) and G/An/Cu (**b**).

In order to further investigate the potential presence of other noncrystalline by-products, hybrid polymers with the highest copper content were analyzed by FTIR spectroscopy. Since Cu is inactive to IR analysis, we can only observe the peaks that can be assigned to the copper compounds, polymeric matrix, and counterions present in the functional groups of the polymer (Figure 2). In the FTIR spectrum, there appear numerous peaks which are indicative for the supporting anion exchanger, whose assignments are presented in Table 4. In accordance with our previous study [36], this analytical technique is not sufficient to strictly identify the copper oxide deposited in the matrix of the hygroscopic polymer, since their characteristic peaks indicative for Cu–O vibrations occur in the range of 500–630 cm^−1^, which is also the frequency of a broad set of the absorption bands of hygroscopic water. However, as a consequence of the reduction of Cu_2_O to Cu, in the spectrum of ZVC-containing hybrid polymer, a characteristic peak of Cu(I)–O vibrations at 630 cm^−1^ disappeared. Although FTIR spectra of the obtained hybrid materials represent the superposition of all components, it is possible to observe bands associated with ascorbic acid—the strong absorption in the range 1710–1775 cm^−1^ attributed to C=O stretching of the lactone ring (Figure 2a,b) [42]. Since the reaction occurs under the excess of ascorbic acid, its dissociated forms are bound as counter ions with the functional groups of the supporting anion exchanger. The presence of ascorbate groups manifested in IR spectra is very beneficial due to their strong antioxidant properties ensuring stability to copper metallic particles during storage [17,43,44]. It can also be observed that after reduction of Cu_2_O into Cu^0^, the area of a broad peak indicative of adsorbed water at 3350 cm^−1^ decreases and almost vanishes in the case of gel-type Cu^0^-containing hybrid polymer. This relation suggests that after the conversion of copper oxide into zero valent copper, the deposit makes the surface of the product less hydrophilic, which is probably due to low chemical activity for water dissociation of the Cu surface, resulting in weak water adsorption properties [45].

#### 3.2.3. Scanning Electron Microscopy Studies

**Figure 3:**
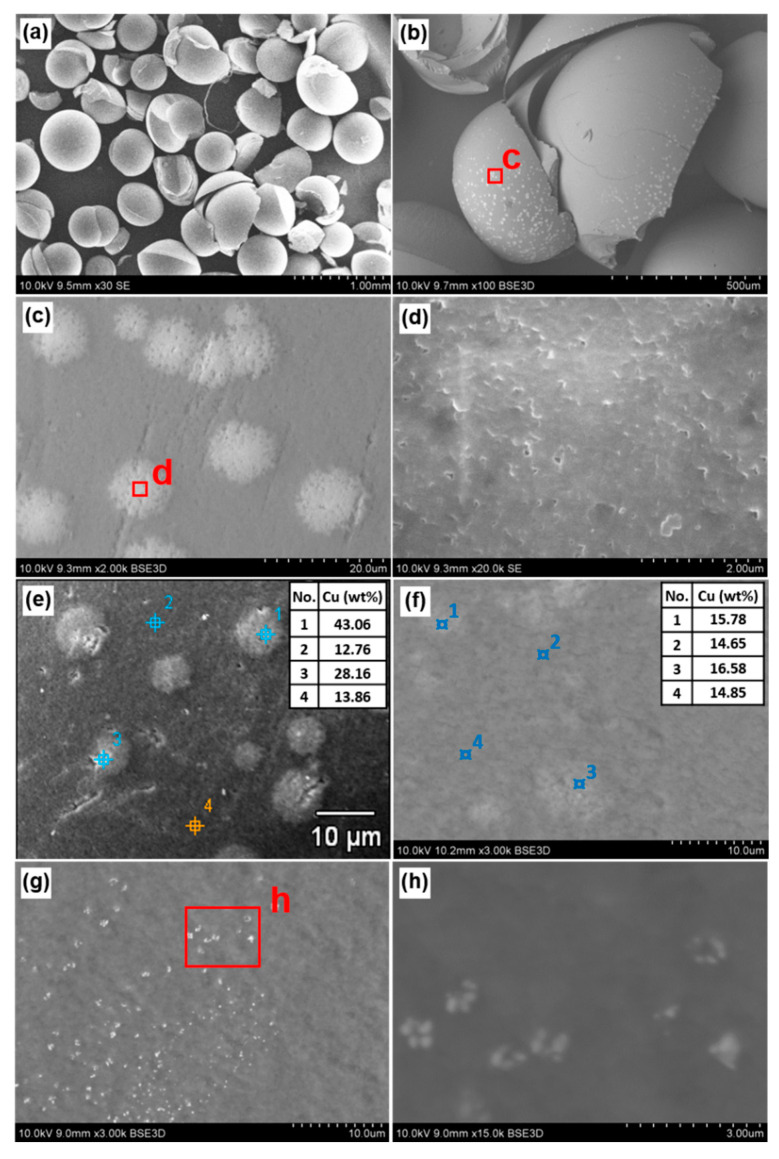
SEM images and EDS analysis of the M/An/Cu (**a**–**e**) surface, (**f**–**h**) inside.

The morphology of the obtained hybrid polymers containing ZVC was studied using scanning electron microscopy. One sample for each type of supporting material, i.e., macroporous and gel-type anion exchanger showing the highest total copper content (in accordance with Table 3), was selected. Thus, among the samples of macroreticular exchangers, the M/An/Cu one obtained at higher temperature and using ascorbic acid with a low pH was tested and for gel exchangers those that were produced at room temperature with almost neutral reducer solution. Figure 3a shows that macroreticular grains are mostly broken. Their external surface is characterized by extensive cracks. Occasionally light spots appear on the surface (Figure 3b–d). According to the EDX (Energy Dispersive X-Ray Analysis), these spot areas contain only copper (Figure 3e). The morphology of these spots differs significantly from the morphology observed for other copper-containing objects. There are no visible particles on the spots but an almost solid layer, which suggests that these areas may be associated with amorphous copper. An EDX analysis, performed on the macroreticular bead cross section, revealed that the copper deposit is distributed throughout the volume of the M/An/Cu grain (Figure 3f). A more careful observation of the interior of the grain reveals a few clusters of closely lying individual particles with a size up to maximum 200 nm (Figure 3g,h).

**Figure 4:**
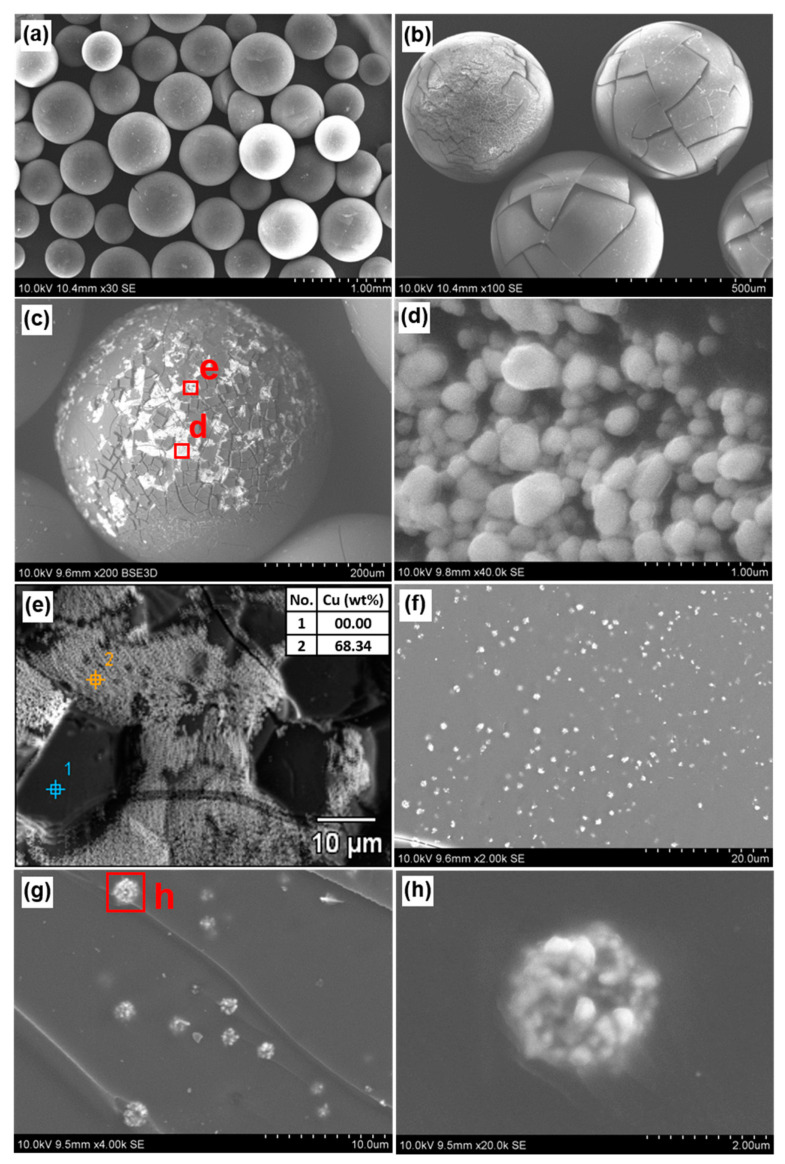
SEM images and EDS analysis of the G/An/Cu (**a**–**e**) surface, (**f**–**h**) inside.

Figure 4 shows the morphology of both the surface (Figure 4a–e) and the interior of the G/An/Cu beads (Figure 4f–h). Gel beads are evidently not broken, but their outer surface is clearly cracked. Extensive cracks are visible on virtually every grain (Figure 4b). In addition, a mesh of minor cracks is observed on some of them (Figure 4c). Higher fracture magnifications reveal numerous copper particles with characteristic hemispherical particle shape and sizes from objects smaller than 100 nm to a maximum of ~500 nm (Figure 4d,e). The interior of the grains is smooth and nonporous with numerous evenly distributed copper aggregates, with a total size of approximately 1 μm, formed from tightly packed particles about 200 nm in size (Figure 4f–h).

Comparing the morphology of the copper deposits obtained in the M/An/Cu and G/An/Cu anion exchangers, it can be seen that the internal structure of the anion exchanger has a decisive impact on the shape and size of the deposit. Our previous research on HIX containing Cu_2_O, used here as a source for the growth of the Cu crystals, also led to similar conclusions [36]. Macroporous beads have permanent pores, in contrast to gel-type grains. Contrary to the pores in G/An, which appear only in the swollen state, the permanent internal macropores in M/An do not collapse when the material loses water, and for this reason they do not lead to dense packing of deposited particles. For this reason, in the M/An matrix no such dense packing of deposited particles was observed as in gel exchangers. In addition to the type of exchanger (its internal structure), the morphology of the Cu_2_O precursor particles undoubtedly had a significant impact on the size and shape of the produced copper metallic particles. In the case of substrate macroreticular M/An/Cu_2_O beads, the Cu_2_O deposit was in the form of evenly dispersed, irregular clusters of a few to 10–20 adjoining spherical objects approximately 200 nm in diameter. After reductive conversion, the resulting copper particles mostly are not visible on SEM images due to their much smaller (compared to the original Cu_2_O) size. Hardly visible copper-containing objects are in the form of clusters of a few particles up to a maximum of 200 nm. In the case of the substrate gel HIX (i.e., G/An/Cu_2_O), this type of matrix induced the formation of compact Cu_2_O aggregates of approximately 1 μm. After reductive conversion, the produced copper particles (about 200 nm in size) no longer form a compact aggregate but rather are loosely connected into a cluster with a diameter of about 1 μm. Apparently, visible changes in the size of Cu^0^ aggregates that were formed after reduction result from a smaller molar volume of copper (7.61 cm^3^/mol) compared to Cu_2_O (23.85 cm^3^/mol) [47]. However, the basic shape of the aggregates formed by nanoparticles is maintained after the reduction process because the initial Cu_2_O nanoparticles are used as a template of transformation for the crystal-to-crystal conversion.

#### 3.2.4. Studies of Porosity

**Figure 5:**
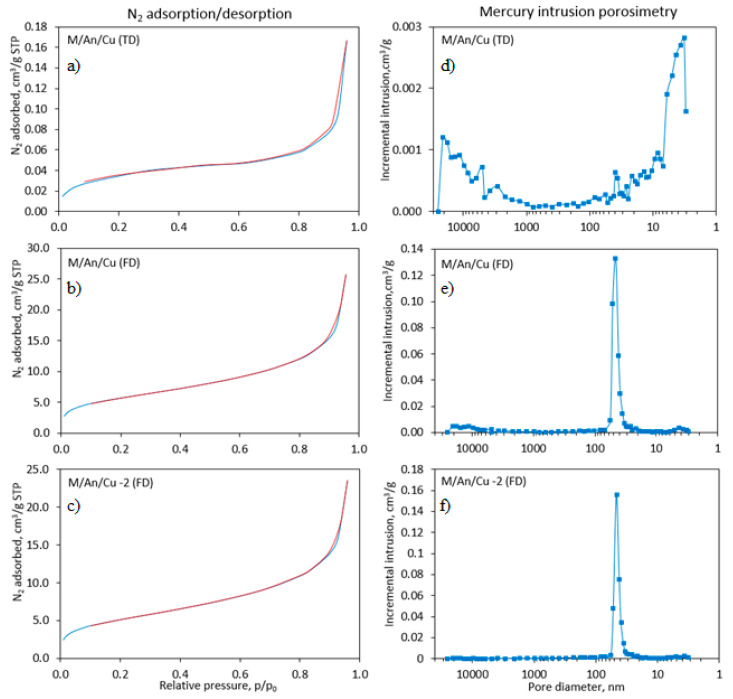
Isotherms of N_2_ adsorption/desorption (**a**–**c**) and pore size distribution determined by mercury intrusion porosimetry (**d**–**f**) for thermally dried M/An/Cu (**a**,**d**) freeze-dried M/An/Cu (**b**,**e**) and freeze-dried M/An/Cu-2 obtained by method-2.

The deposition of ZVC load in the multistep process including drying and wetting in aqueous solutions by the shrink-swell mechanism may affect not only the physical integrity of the beads but also their porous structure. As can be seen in Figure 3a, showing the products obtained by pouring a dry An/Cu_2_O sample into the solution, due to the rapid swelling of the ion exchanger, the beads, particularly the M/An/Cu ones, are cracked. This observation is also reflected in changes of macroporous structure of the thermally dried polymeric beads after Cu^0^ deposition. A comparison of pore size distribution determined using the mercury intrusion method for the thermally dried product (M/An/Cu) with those obtained for the thermally dried initial anion exchanger (M/An) [48] shows that after ZVC incorporation, macropores with a diameter exceeding 1 µm appeared, and the pore size distribution changed from uniform to wide-ranging dimensions. This change is probably a result of resin structure disintegration, since aggregates of Cu^0^ deposited within pores of the supporting material may influence its resistance to osmotic pressure [49]. A further analysis of porous parameters (Table 5)—BET (Brunauer, Emmett and Teller) surface area (micro and mesoporous structure) and porosity determined by mercury intrusion (macro- and mesoporous structure)—which decreased significantly after Cu^0^ deposition from 21.7 m^2^/g to almost 0.0 m^2^/g and from 29.8 to 4.7%, respectively, confirm the blockage and closure of porous structure of the host polymer with aggregates of the inorganic constituent. Additionally, the effect of pore blockage may be amplified by copper aggregation and its more hydrophobic nature, resulting in less hygroscopic water content and a less swollen structure of the polymer, which is in agreement with the results of the FTIR analysis. In consequence, lower hydration, screening of pore walls, squeezing, and structure defects due to the presence of the inorganic deposit may lead to the shrinkage and collapse of the structure of the transport macropores, which is reflected in the more significant loss in the porosity and increase in apparent density. Simultaneously, the shape of the N_2_ adsorption–desorption isotherm—type IV—and its hysteresis loop—type H1—of all studied materials is indicative for the presence of cylindrical mesopores, even in the case of thermally dried M/An/Cu, which showed a very small BET surface area (Figure 5a,b). This supports our conclusion that Cu^0^ aggregates completely close the greater part of mesopores in the matrix of the dried anion exchanger. Since the gel-type ion exchangers in a dry state do not possess any internal surface area, the porous structure of gel-type products was not studied. During drying, their structure shrinks and the plot of the isotherm is different—it is a straight line with no hysteresis loop—indicating that the N_2_ adsorption occurs only on the outer surface of the beads.

Unlike thermal drying, freeze drying of the hybrid anion exchangers allowed their porous structure to be preserved even in a dried state. The macroporosity did not change after Cu^0^ deposition (Table 5), and the pore size distribution that was determined by mercury intrusion was very narrow, indicating the presence of mesopores with the maximum at 40 nm in the case of both the virgin supporting polymer [48,50] and the ZVC-containing hybrid product (Figure 5e,f). After Cu deposition, we observed a small volume increase in the range of macropores with dimensions exceeding 5 µm, which may be an effect of insignificant disintegration of the polymeric beads. The mesoporous structure of the beads was more significantly affected. The mean pore diameter determined from the N_2_ adsorption isotherm increased from 4.4 to 7.7 nm while the BET surface area decreased from 27.8 m^2^/g to 20.8 m^2^/g, which probably resulted from the blockage of the smallest pores of the anion exchanger. Summing up, the method of drying is crucial to the mechanical resistance of the obtained materials while the changes in porous characteristics may be reversible due to the elastic structure of the polymeric matrix in aqueous solutions (by swelling in electrolyte solution and the subsequent reopening of the porous polymeric structure). Nevertheless, freeze drying seems to be a significantly better method when the application of the obtained material involves gaseous reagents. In turn, thermal drying resulting in the collapse of porous structure may restrict gas permeation and consequently the oxidation of ZVC load during storage in atmospheric air.

### 3.3. Operational Control of Physical Form and Copper Deposit Crystallinity of Hybrid Anion Exchangers

**Figure 6:**
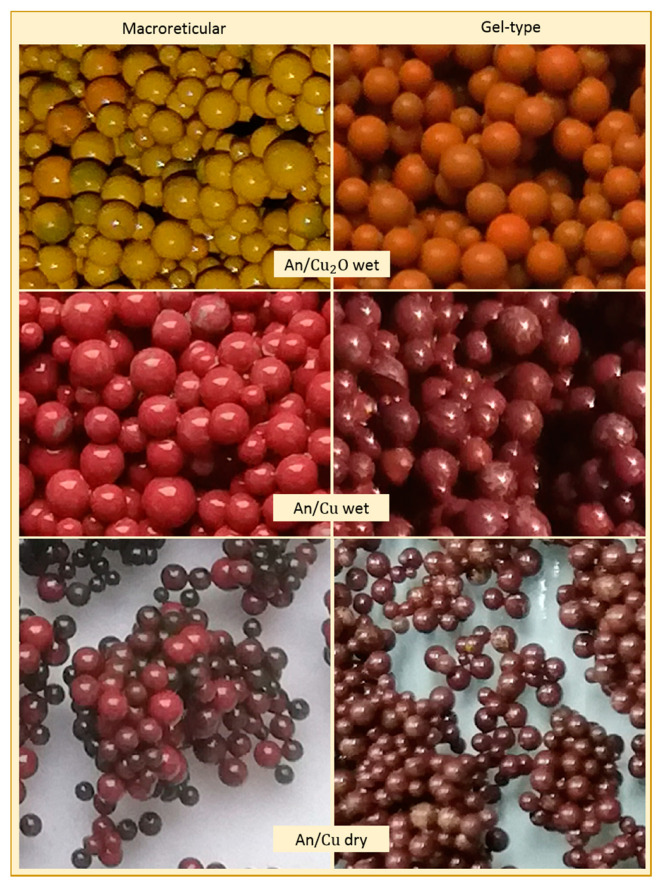
Photographs of examined materials: based on macroporous anion exchanger (left column) and gel-type anion exchanger (right column).

In order to avoid the shrinkage and swelling of the polymeric carrier, the synthesis was modified by introducing a vacuum-filtered (wet) An/Cu_2_O sample into the reaction medium (Table 3, Method 2). Taking into consideration the previously established fact that the reaction rate is higher in the AA solution alone, only this solution and a shorter reaction time were used in the next stage of the research. Almost immediately after they had been introduced into the reaction medium, both the samples changed their color to claret, no sediment was observed in the aqueous phase, and the reaction products had uniform, undamaged grains (see the photos in Figure 6).

**Figure 7:**
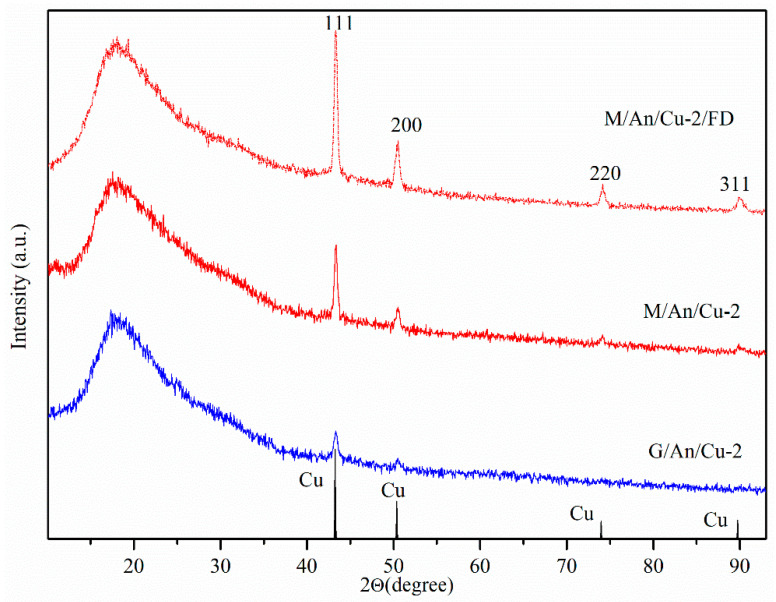
XRD patterns of reduction products M/An/Cu-2 and G/An/Cu-2 obtained by Method 2.

An XRD analysis (Figure 7) confirmed the assumption that the short reaction time (30 min) was sufficient in order to convert Cu_2_O into Cu^0^ in both the macroreticular and gel-like anion exchanger. In the case of the latter product, this result evidently showed that the swelling of the polymeric matrix significantly influences the kinetics of the reduction process (Table 3). A comparison of XRD diagrams of samples obtained by Method 1 (Figure 1, second line from the top in M/An/Cu and G/An/Cu part), with diffractograms of samples from Method 2 (Figure 7, M/An/Cu-2 and G/An/Cu-2) shows that the $\frac{I_{amorf}}{I_{\left(111\right)Cu}}$ ratios in the diagrams from Method 2 are clearly greater than in the case of the corresponding samples obtained by Method 1, which indicates a reduced fraction of crystalline Cu^0^ in these samples.

**Figure 8:**
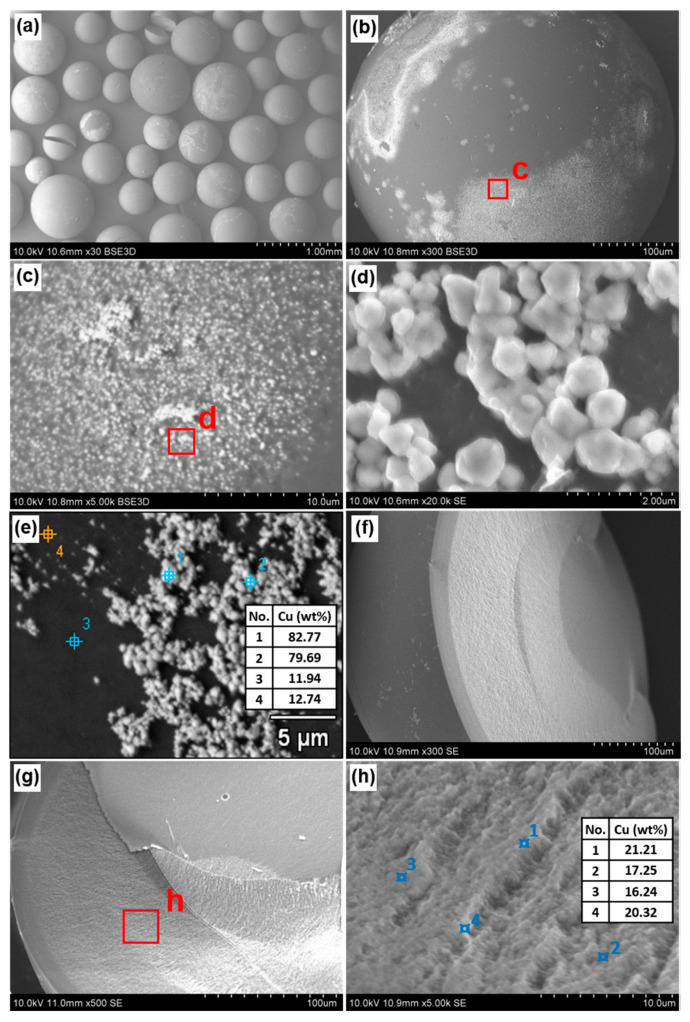
SEM images and EDS analysis of the M/An/Cu-2 (**a**–**e**) surface, (**f**–**h**) inside.

**Figure 9:**
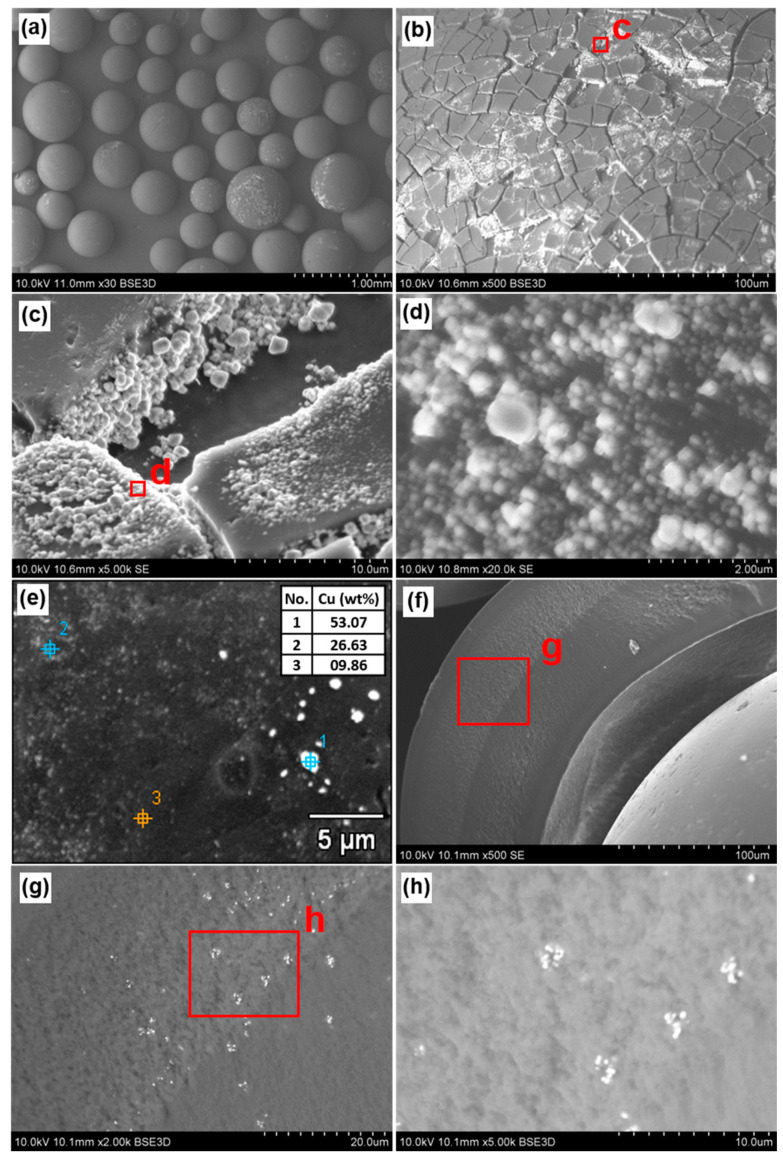
SEM images and EDS analysis of the G/An/Cu-2 (**a**–**e**) surface, (**f**–**h**) inside.

As might be expected, Method 2 in the case of macroreticular exchangers made it possible to prevent grain breakage. The M/An/Cu-2 (2 signifies Method 2) grains are undisturbed (Figure 8), and on their outer surface characteristic white spots are visible, identified as copper-rich areas (Figure 8a–e). The magnification of these areas reveals particles with a size and shape reminiscent of those previously identified on the surface of the G/An/Cu-1 (1 signifies Method 1) beads (Figure 8d). There are no characteristic bright objects inside the beads that would indicate the presence of copper-containing particles. The EDX analysis reveals that copper is dispersed equally in their entire volume (Figure 8h). Figure 9 presents a microscopic analysis of the surface and interior of the G/An/Cu-2 exchanger. In contrast to grains of the same anion exchangers obtained by Method 1 (G/An/Cu-1), grains obtained by a modified Method 2 do not show extensive cracks on the outer surface. Only some beads exhibit, as in Method 1, a mesh of small cracks on the surface (Figure 9a,b). An enlarged image of these cracked areas reveals numerous copper particles of the same shape and size as in the case of Method 1 (Figure 9c–e). As in the case of G/An/Cu-1, numerous copper aggregates with a size of about 1 μm are visible on the cross-section of G/An/Cu-2 grains (Figure 9f–h). However, in this case, the aggregates are formed of loosely packed particles with a size of about 200 nm. Evidently, because in the first method the G/An/Cu_2_O samples were dried before the next reduction step, the matrix swelling and then its collapse caused more packing of the resulting copper deposit in G/An/Cu-1. Skipping the drying stage resulted in the lack of shrinkage and a much less packed distribution of the resulting copper particles in the G/An/Cu-2 beads (Figure 9g,h).

**Figure 10:**
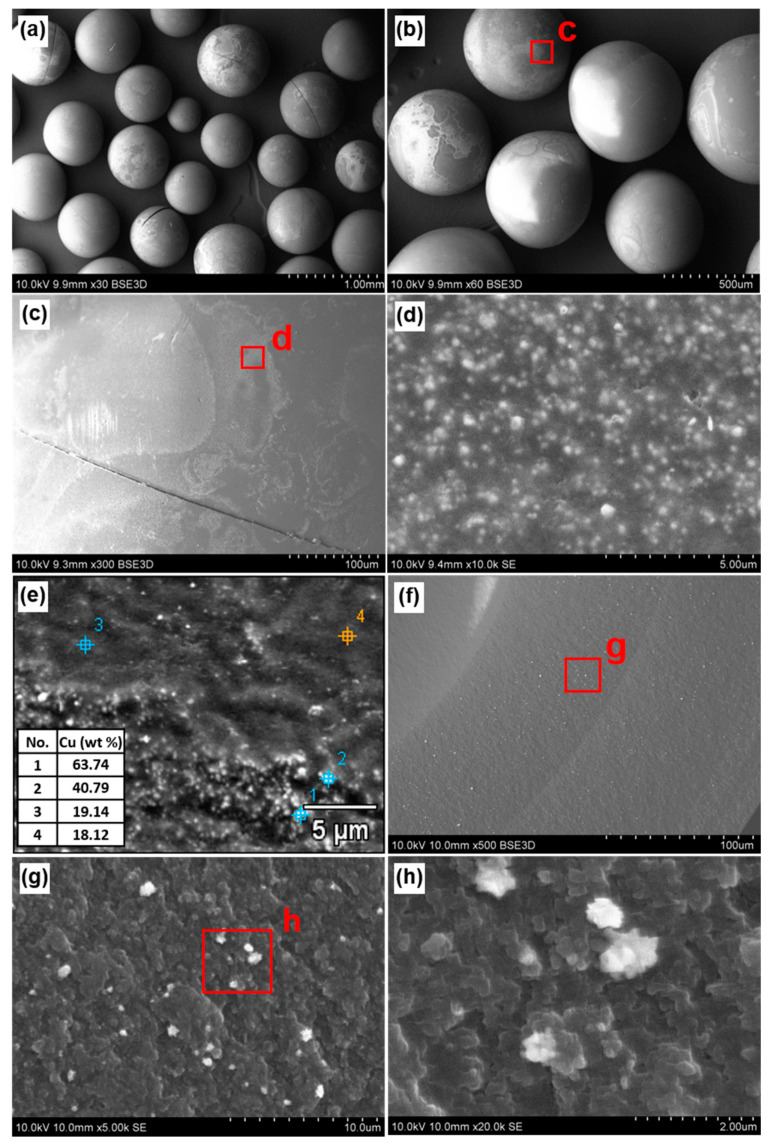
SEM images and EDS analysis of the M/An/Cu-2/FD (**a**–**e**) surface, (**f**–**h**) inside.

To avoid significant deformation and eliminate the shrinkage or toughening of the macroreticular M/An/Cu HIX beads resulting from the drying stage, the samples obtained by Method 2, instead of being conventionally dried after the reduction, were also freeze dried. Figure 10 shows photographs of the surface and interior of M/An/Cu-2 beads after freeze drying. On the surface of the macroreticular exchanger beads after lyophilization, numerous white areas can be observed, the magnification of which reveals numerous fine copper particles (Figure 10a–c). These copper particles appear to be more separated after freeze drying (Figure 10d,e) than what is observed for conventionally dried M/An/Cu-2 samples. Evenly dispersed white objects are visible on the cross-section images of freeze-dried grains (Figure 10f–h). These asymmetrical, star-like objects contain copper, and their size can be approximately estimated to be 0.2–1 μm (Figure 10h).

On the cross-section images of the conventionally dried M/An/Cu-2 beads, copper particles were not visible. Clearly, the M/An/Cu-2 drying method affects the shape and size of the deposit. Lyophilization involves two stages, namely freezing, in which freezing water forms ice crystals, and drying, when a vacuum is applied to sublimate the ice. Recent studies on nanoparticle lyophilization have shown that this method of drying causes different stresses during both stages of the process, contributing to an increased aggregation and agglomeration of nanoparticles [51,52]. Apparently, the growth of ice crystals in the pores of the M/An/Cu-2 macroreticular exchanger affected the deposit structure, resulting in the formation of large crystalline copper particles. The crystallization of ice caused mechanical stress on copper deposited in pores, leading not only to a denser packing of the deposit but also to an increase in the degree of its crystallization. A higher content of crystalline copper in freeze-dried samples was confirmed by XRD measurements (see the M/An/Cu-2/FD line in Figure 7). The analysis of porous characteristics of M/An/Cu-2 showed slightly lower porosity in the region of the macropores and also a very narrow pore size distribution in the range of the mesopores in comparison with the previous sample (M/An/Cu/FD) (Table 5, Figure 5c,f). The implication of this result is that elimination of operations conducted under elevated temperatures during Cu^0^ deposition preserves the mechanical resistance and uniformity of porous characteristics of the host anion exchanger [48]. Simultaneously, larger crystalline Cu particles formed during lyophilization caused a slight decrease in BET surface area and an insignificant increase in mean pore diameter.

## 4. Conclusions

Fine Cu_2_O particles dispersed in the anion exchanger phase can be easily transformed into Cu^0^ particles, which after the reaction remain in the anion exchanger phase. The transformation proceeds fairly quantitatively in the matrix of both the macroreticular anion exchanger and the gel-like anion exchanger. Ascorbic acid (an ecofriendly reducing agent) is the reagent that makes this transformation possible. The reduction also proceeds in a sodium ascorbate solution but at a slower rate, despite the fact that the redox potential of this solution is lower than that of the ascorbic acid solution. A major benefit resulting from the use of ascorbic acid is that during reduction, the anion exchanger’s functional groups are transformed into the ascorbate form, thereby protecting the metallic copper particle against oxidation (ascorbic acid is a well-known antioxidant). Having such substrates as preformed M/An/Cu_2_O and G/An/Cu_2_O (rich in the copper precursor) as templates for reductive conversion of Cu_2_O to Cu^0^, we managed in mild conditions to obtain HIXs with 7.0 and 5.0 wt% of the total Cu content.

While the conditions of the reduction reaction influenced the kinetics and efficiency of the process, as shown by the SEM EDS analyses, the morphology and distribution of copper within the structure of polymeric beads were mainly determined by the type of porosity of the supporting polymer and the distribution of preformed Cu_2_O particles in the beads of the starting material. The G/An/Cu matrix contained loosely connected copper particles that formed clusters about 1 μm in size, whereas M/An/Cu beads contained clusters of closely lying individual particles with a size up to a maximum of 200 nm. In turn, the porous structure and integrity of the beads were dependent on the procedure of the whole process of the drying operations before and after the reduction reaction. The best products were obtained by using wet M/An/Cu_2_O or G/An/Cu_2_O as a starting material, conducting the reduction over 30 min at 50 °C, under acidic pH and freeze drying the final product. This procedure enabled us to obtain material with uniformly distributed star-like particles of ZVC with a high degree of crystallization and simultaneously with a preserved porous structure of the virgin supporting anion exchanger. In contrast, thermally dried products may exhibit a higher resistance for oxidation due to collapsed porous structure resulting lower gas permeability. Considering that both reaction parameters and operational techniques influence the size, shape, and distribution of Cu^0^, as well as the porous structure of polymeric matrix, the obtained composite materials may have promising applications, including chemical catalytic or photocatalytic reactions involving both gaseous or liquid reagents as well as antibacterial activity.

## Figures and Tables

**Table 1:** Reducer solutions.

Symbol of Solution	Reducing Agent Solution	Potential (mV)	pH
AA/2	1 M ascorbic acid	+113	2.12
AA/6	1 M ascorbic acid in 1 M NaOH	−195	6.42

**Table 2:** Hybrid ion exchangers with Cu_2_O deposit.

Properties	Symbol of HIX	
	M/An/Cu_2_O	G/An/Cu_2_O
Polymeric carrier	Amberlite IRA 900Cl	Amberlite IRA 402OH
Physical form	Spherical beads	Spherical beads
Polymer matrix	Styrene/divinylbenzene copolymer	Styrene/divinylbenzene copolymer
Functional groups	Trimethylammonium	Trimethylammonium
Matrix structure	Macroreticular	Gel-like
Inorganic deposit	Cu_2_O	Cu_2_O
Cu content in HIX, wt%	8.6	6.6
Appearance of dispersed Cu_2_O deposit	Unsymmetrical bracelet-like clusters of a few to 10–20 adjoining spherical objects approximately 200 nm in diameter	Highly symmetrical, single, separate, ideally shaped spheres approximately 1.0 μm in diameter

**Table 3:** Conditions and results of Cu_2_O reduction.

Symbol of HIX	Solution	Temp. (°C)	Reaction Time (h)	Cu Content in An/Cu (wt%)
*Method 1, dry Cu_2_O as a precursor of Cu*				
M/An/Cu_2_O	AA/2	20	24	6.76
		50	3	7.68
	AA/6	20	24	6.65
		50	3	6.83
G/An/Cu_2_O	AA/2	20	24	4.53
		50	3	4.50
	AA/6	20	24	5.35
		50	3	5.32
*Method 2, wet Cu_2_O as a precursor of Cu*				
M/An/Cu_2_O	AA/2	50	0.5	6.90
G/An/Cu_2_O				5.10

**Table 4:** Assignments of infrared bands of the host anion exchangers [46].

Assignment	Wavenumber cm^−1^
O–H vibrations of hygroscopic water	1650, 3100–3600
C=C and C–H vibrations of benzene rings and aliphatic groups	1020, 1111, 1418, 1478, 1512, 1613, 3020
C–H, C–N, CCN vibrations in CH_3_–N	707, 826, 859, 889, 926, 975, 1221, 1341, 1383, 2855, 2922

**Table 5:** Porous characteristics of supporting anion exchanger and Cu^0^-doped anion exchanger (additional sample specification FD means freeze-dried samples).

Sample Code	S_BET_^a^ (m^2^/g)	V_T_ (Pore Volume) ^a^ (cm^3^/g)	L_m_ (Pore Diameter) ^a^ (nm)	Total Surface Area ^b^ (m^2^/g)	Total Intrusion Volume ^b^ (cm^3^/g)	Porosity ^b^ (%)	Bulk Density (g/cm^3^)
Amberlite IRA 900Cl *							
M/An	21.7	0.0544	5.0	60.95	0.3945	29.84	0.877
M/An/FD	27.8	0.0607	4.4	56.16	0.5195	37.17	0.716
*Product of Method 1*							
M/An/Cu	0.13	0.001	Indeterminable	16.3	0.0364	4.68	1.288
M/An/Cu/FD	20.8	0.040	7.7	46.2	0.443	37.3	0.842
*Product of Method 2*							
M/An/Cu-2/FD	18.4	0.037	7.9	43.5	0.390	34.6	0.886

^a^ N_2_ adsorption/desorption measurement, ^b^ mercury intrusion porosimetry, * results from previous studies [48].
